# Sex Proportionality in Pre-clinical and Clinical Trials: An Evaluation of 22 Marketing Authorization Application Dossiers Submitted to the European Medicines Agency

**DOI:** 10.3389/fmed.2021.643028

**Published:** 2021-03-11

**Authors:** Marieke J. H. J. Dekker, Sieta T. de Vries, Carolien H. M. Versantvoort, Ellen G. E. Drost-van Velze, Mansi Bhatt, Peter J. K. van Meer, Ineke K. Havinga, Christine C. Gispen-de Wied, Peter G. M. Mol

**Affiliations:** ^1^Dutch Medicines Evaluation Board, Utrecht, Netherlands; ^2^Department of Clinical Pharmacy and Pharmacology, University of Groningen, University Medical Centre Groningen, Groningen, Netherlands

**Keywords:** clinical trials, sex, sex distribution, proportionality, disease prevalence, efficacy, safety, subgroup analysis

## Abstract

This study assessed to what extent women were included in all phases of drug development; whether the clinical studies in the marketing authorization application dossiers include information per sex; and explored whether there are differences between women and men in the drugs' efficacy and safety. Data were extracted from dossiers submitted to the European Medicines Agency. Twenty-two dossiers of drugs approved between 2011 and 2015 for the treatment of various diseases were included. Female animals were included in only 9% of the pharmacodynamics studies, but female and male animals were included in all toxicology studies. Although fewer women than men were included in the clinical studies used to evaluate pharmacokinetics (PK) (29 to 40% women), all dossiers contained sex-specific PK parameter estimations. In the phase III trials, inclusion of women was proportional to disease prevalence for depression, epilepsy, thrombosis, and diabetes [participation to prevalence ratio (PPR) range: 0.91–1.04], but women were considered underrepresented for schizophrenia, hepatitis C, hypercholesterolemia, HIV, and heart failure (PPR range: 0.49-0.74). All dossiers contained sex-specific subgroup analyses of efficacy and safety. There seemed to be higher efficacy for women in one dossier and a trend toward lower efficacy in another dossier. More women had adverse events in both treatment (73.0 vs. 70.6%, *p* < 0.001) and placebo groups (69.5 vs. 65.5%, *p* < 0.001). In conclusion, women were included throughout all phases of clinical drug research, and sex-specific information was available in the evaluated dossiers. The included number of women was, however, not always proportional to disease prevalence rates.

## Introduction

“How excluding women from clinical trials is hurting our health” (1) and “Most biomedical studies fail to report if results differ by sex” (2) are just two newspaper headings, exemplifying the large body of media attention suggesting that women are underrepresented in drug trials and, if included, that the data are not analyzed and/or reported for women separately. These concerns are based on findings of several studies assessing sex proportionality in pre-clinical or clinical research (3–5). However, it has been argued that the underrepresentation of women has improved over the years (6–8). This improvement follows changes in regulatory requirements over time, where coming from an era in which there was great reluctance to include women in clinical trials after the thalidomide disaster, societal pressure made the Food and Drug Association (FDA) change their position in 1993 to recommend inclusion of more women in clinical trials (9). A recent study evaluating FDA's publically available drug registration dossiers of commonly prescribed drugs, indeed rejected any systematic underrepresentation of women in clinical trials and suggested that some type of sex-specific analysis has been performed in most cases (10).

A 2005 review by global regulatory authorities concluded that phase I and II clinical trials were slightly underrepresented with respect to women, but not the confirmatory phase III trials (11). The population in phase III trials should equate disease prevalence in women and men to reflect as much as possible the real world population in a controlled setting (12). Not recruiting a transposable population for this trial phase may result in a biased understanding of drug effects, benefits and harms, in the real world target population (9, 13). Previous studies have also shown a larger sex disproportionality in the early trial phases compared to the later trial phases and suggest that there may be differences in sex proportionality across disease areas (10, 14).

Currently, representation of women has not been assessed for each phase in the drug development process using data directly from the marketing authorization application (MAA) dossiers. These dossiers are, however, the source for regulators to decide about the marketing authorization of drugs and are far more detailed than what is ultimately published on regulators' websites in their public assessment reports. Therefore, the aim of this study was to assess to what extent women were included in all phases of drug development, that is in preclinical animal studies, clinical studies evaluating pharmacokinetics (PK) from phase I to phase III, and—proportionally to disease prevalence—in the phase III clinical trials, for various diseases using the information in the MAA dossiers. Our secondary aim was to assess whether the clinical studies in the MAA dossiers include efficacy and safety information per sex and to explore whether there are differences between women and men in the drugs' efficacy and safety.

## Methods

Data were extracted from MAA dossiers at the Dutch Medicines Evaluation Board. These dossiers follow a globally standardized format, i.e., Common Technical Document (15), and contain thousands of pages with administrative data up to the smallest detail of trial data. The standardized format consists of five modules with Module 1 containing region-specific administrative information, and Modules 2–5 containing information common for all regions about quality (Module 3), non-clinical study reports (Module 4), clinical study reports (Module 5), and a summary and overview of these aspects (Module 2).

Included were the dossiers of a sample of drugs submitted for marketing authorization through centralized procedures to the European Medicines Agency (EMA) and approved for marketing authorization between 2011 and 2015—i.e., the most recent five years when we initiated this study—for the treatment of nine major indications in three disease areas; (1) infectious diseases; hepatitis C, human immunodeficiency virus (HIV), (2) central nervous system diseases; depression, schizophrenia, epilepsy, and (3) cardio metabolic diseases; heart failure, thrombosis, diabetes mellitus, and hypercholesterolemia. These diseases were selected because there were a number of drugs approved in recent years, and/or there was a suggestion of poor representation of women in clinical trials or there were possible sex differences in effects (16). We excluded dossiers that were not based on a full (or complete) dossier (article 8.3) (17), as only full dossier applications contained the comprehensive set of data on the pharmaceutical development, non-clinical studies (pharmacological and toxicological), and clinical trials, including PK studies needed to perform our review.

We reviewed data on sex representation in pre-clinical animal studies, clinical studies evaluating PK from phase I to phase III including population PK studies and sex distribution and proportionality, i.e., representation of women in relation to the disease prevalence, in the phase III clinical trials. In addition, we assessed whether reported drug effects in the clinical PK evaluations and phase III clinical trials were presented and/or described per sex and whether efficacy and safety data suggest sex differences.

### Sex Assessment in Pre-clinical Studies

The sex of included animals was extracted from the pharmacodynamics (PD) and the toxicology animal studies. The first type of animal studies are performed to support the efficacy of the drug in the target indication and provide an understanding of the mechanism of action. The second are standard International Conference on Harmonization (ICH)-defined studies to understand basic toxicology of a new drug product. Information was extracted from Module 2 and where necessary from Module 4 of the dossiers.

### Sex Assessment in Clinical Studies Evaluating Pharmacokinetics

From the complete evaluation of PK in phase I, phase II and III trials, and the population-PK studies included in Module 2 and where necessary Module 5 of the dossiers, the percentage of included women was determined per study. Next, we assessed whether the key PK parameters, that is area under the curve (AUC, a measure for drug exposure) and maximum concentration (Cmax), were presented per sex. In case no AUC and Cmax were provided, we evaluated which other PK-measures were presented per sex.

### Sex Assessment in Phase III Clinical Trials

From the phase III clinical trials included in Module 2 and where necessary Module 5 of the dossiers, the number of participants and the sex distribution was extracted. Additionally, we assessed whether efficacy and safety subgroup analyses by sex were included, whether the efficacy was different between women and men, and we collected the number of adverse events (AEs) separately for women and men for both the treatment and placebo groups. In case the number of AEs per sex was not available, we evaluated whether other sex-specific safety measures such as serious AEs or AEs of specific interest were available.

### Analyses

We assessed how many studies included male animals only, female animals only, both male and female animals, or did not mention the sex of the animals. This was calculated for the total sample and per disease.

For the clinical studies evaluating PK, we calculated the mean percentage of women and men included in the different phases (i.e., phase I, phase II and III, and population PK studies). Per disease, women to men ratios were calculated for the mean AUC and Cmax. A 0.8 to 1.25 exposure rate was interpreted as a non-relevant difference, as this is the range considered acceptable for demonstrating bioequivalence between drug formulations in generic applications by the EMA (18).

For the phase III clinical trials, the overall number of women and men included was calculated. Proportionality of the sex distribution was determined by calculating the participation to prevalence ratio (PPR) (19, 20) in which the percentage of women in the studies was divided by the percentage of women in the disease population. Data about disease prevalence rates in Europe per sex were obtained from the Global Health Data Exchange (http://ghdx.healthdata.org/) using data from the year 2010. For thrombosis, prevalence data were not available in this database. For this, a scientific publication was used of prevalence data of total hip (THR) and knee replacements (TKR) in the US (21), since the phase III studies of this MAA were conducted among patients undergoing THR and TKR. A ratio between 0.8 and 1.2 was considered as proportional with a representation of women in the studies similar to the representation of women in the disease population, whereas, a ratio <0.8 or >1.2 was considered, respectively an underrepresentation or overrepresentation of women in the studies (19, 20).

Descriptive statistics were used to assess the number of dossiers that contained sex-specific information on efficacy and safety. We additionally calculated women to men ratios for the efficacy parameter assessed for each of the dossiers using placebo-adjusted data (e.g. odds ratios, mean difference to placebo), active-comparator-adjusted data in case of a preventive drug (primary or secondary prevention), or descriptive changes (e.g. percentages, mean change from baseline) in case of missing placebo-adjusted data. For the safety, the mean percentage of women and men experiencing at least one AE for the drug and placebo groups was calculated per drug and overall. Differences in the number of women and men having AEs for the drug and placebo groups were calculated using Chi-squared tests. *P*-values < 0.05 were considered statistically significant.

All analyses were conducted using Microsoft Excel® version 2010.

## Results

**Figure 1:**
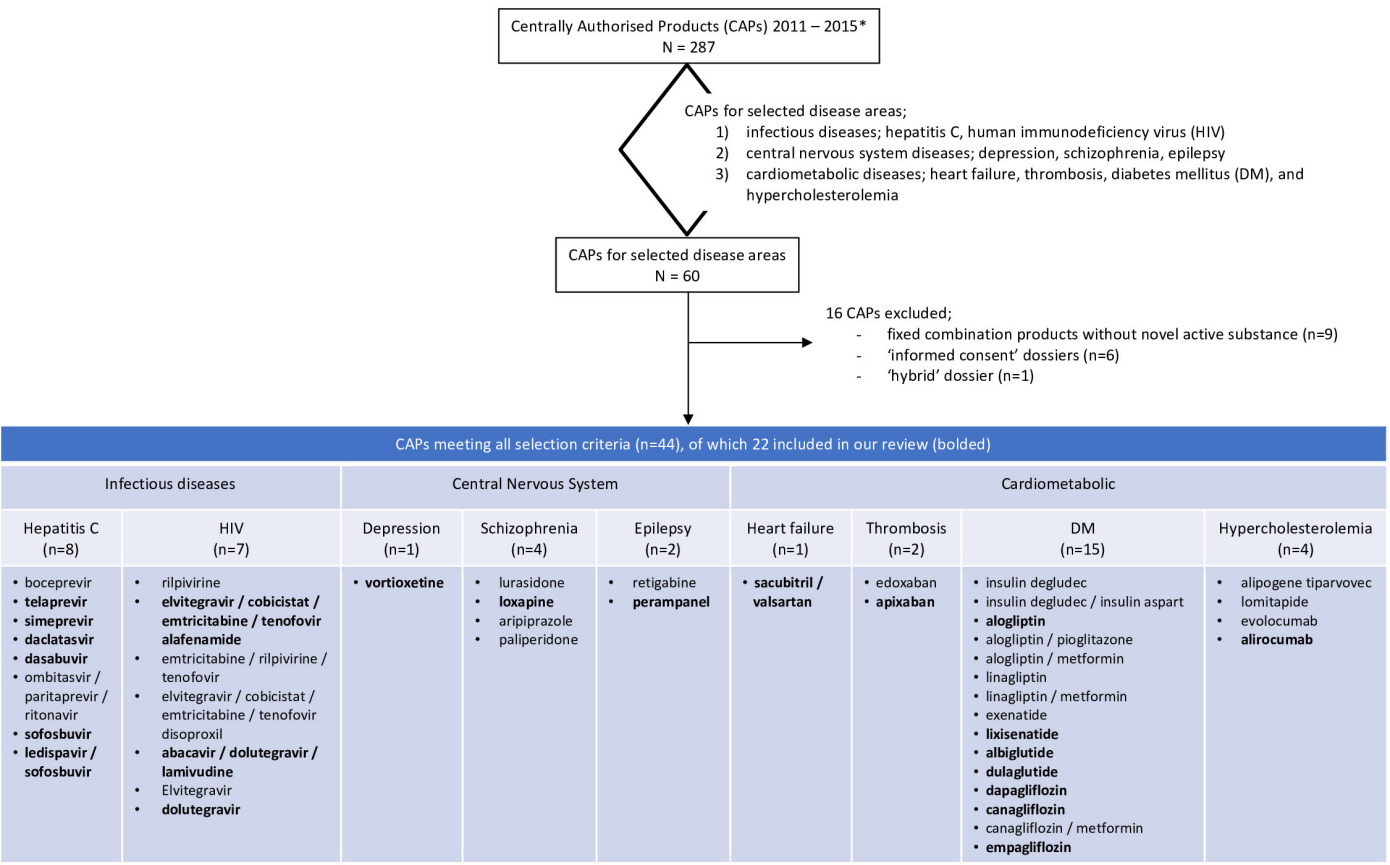
Flowchart of the included dossiers. ^*^Information extracted from https://www.ema.europa.eu/en/medicines/download-medicine-data.

In total, 287 medicinal products were centrally approved in the European Union between Jan 1, 2011 and Dec 31, 2015. Sixty of these 287 products were for the treatment of one of the nine indications selected for our review. We excluded 16 of these dossiers, because these applications were not based on a full dossier (article 8.3); i.e., nine fixed combination products without novel active substance, six “informed consent” dossiers referring to another approved product, and one “hybrid” dossier. Of the 44 dossiers fulfilling our study criteria we included half in our review since it was not feasible to evaluate all 44 dossiers. The dossiers were randomly selected per disease which resulted in the inclusion of 22 dossier of which seven were for drugs to treat diabetes mellitus, six for hepatitis C, three for HIV, and one each for depression, schizophrenia, epilepsy, heart failure, thrombosis, and hypercholesterolemia (Figure 1).

### Sex Assessment in Pre-clinical Studies

For eleven of the 22 dossiers, 124 mechanistic *in-vivo* PD animals studies were available (Table 1). These studies included male animals only, female animals only, both, or did not mention the sex of the animals in respectively 86, 5, 4, and 5% of the studies. There were no clear differences in these percentages across the diseases. All 22 dossiers contained toxicology studies and all included both female and male animals (Table 1). Female animals were included in all conventional non-clinical toxicology programs in accordance with ICH Safety guidelines (www.ich.org), and e.g., to evaluate the impact of drugs on reproductive toxicity (ICH Reproductive Toxicity guideline S5) and in juvenile animals to investigate the drug's impact on e.g., sexual development (ICH Non-clinical Pediatric Safety guideline S11).

**Table 1:** Inclusion of female and male animals in the pre-clinical pharmacodynamics and toxicology studies in 22 Marketing Authorization Application (MAA) dossiers.

**Disease**	**MAA dossier (*N* = 22)**	**Pharmacodynamics studies**					**Toxicology studies**
		**Use of animals**	**Male animals only, *N* (%)**	**Female animals only, *N* (%)**	**Both male and female animals, *N* (%)**	**Sex not mentioned, *N* (%)**	**Female animals used**
Hepatitis C	daclatasvir	No					Yes
	dasabuvir	No					Yes
	sofosbuvir	No					Yes
	simeprevir	No					Yes
	telaprevir	No					Yes
	sofosbuvir/ledipasvir	No					Yes
HIV	elvitegravir/cobicistat/emtricitabine/ tenofovir alafenamide	No					Yes
	dolutegravir	No					Yes
	dolutegravir/abacavir/lamivudine	No					Yes
Depression	vortioxetine	Yes	14 (100)	0 (-)	0 (-)	0 (-)	Yes
Schizophrenia	loxapine	No					Yes
Epilepsy	perampanel	Yes	12 (71)	2 (12)	2 (12)	1 (6)	Yes
Heart failure	sacubitril/valsartan	Yes	14 (93)	0 (-)	0 (-)	1 (7)	Yes
Thrombosis	apixaban	Yes	3 (100)	0 (-)	0 (-)	0 (-)	Yes
Hypercholes-terolemia	alirocumab	Yes	7 (78)	1 (11)	1 (11)	0 (-)	Yes
Diabetes mellitus	albiglutide	Yes	11 (65)	0 (-)	2 (12)	4 (24)	Yes
	empagliflozin	Yes	3 (75)	1 (25)	0 (-)	0 (-)	Yes
	dulaglutide	Yes	5 (100)	0 (-)	0 (-)	0 (-)	Yes
	dapagliflozin	Yes	9 (100)	0 (-)	0 (-)	0 (-)	Yes
	canagliflozin	No					Yes
	lixisenatide	Yes	10 (100)	0 (-)	0 (-)	0 (-)	Yes
	alogliptin	Yes	19 (90)	2 (10)	0 (-)	0 (-)	Yes
**Total**			**107 (86)**	**6 (5)**	**5 (4)**	**6 (5)**	

### Sex Assessment in Clinical Studies Evaluating Pharmacokinetics

**Figure 2:**
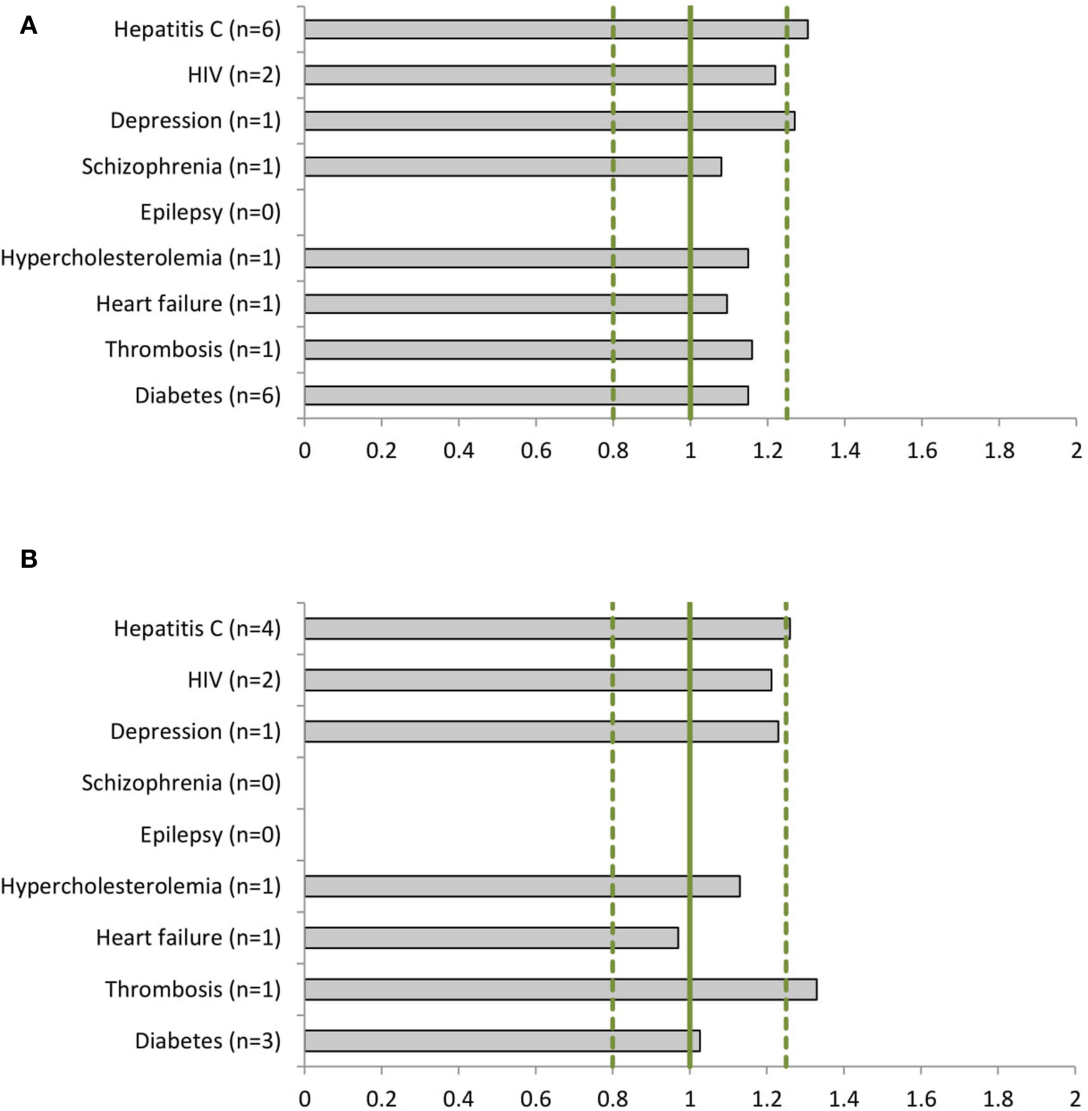
Women-to-men ratios of **(A)** the area under the curve (AUC) and **(B)** the maximum concentration (Cmax) per disease (number of dossiers included). For epilepsy there are no AUC or Cmax data available. The clinical dossier of perampanel used population pharmacokinetics (PK) to estimate the sex impact on clearance parameters. For schizophrenia only total exposure (AUC) data have been reported for women.

We identified 556 phase I, 120 phase II and III clinical studies and 60 population PK studies in which PK was evaluated, including an average of 29, 36, and 40% women, respectively, in the 22 dossiers (Table 2). All dossiers contained sex-specific information on PK parameters. In women, total exposure (AUC) ranged from 1.08-fold (schizophrenia) to 1.30-fold (hepatitis C) higher than in men (Figure 2A). Similarly, the rate of exposure (Cmax) ranged from 0.97-fold (heart failure) to 1.33-fold (thrombosis) higher (Figure 2B). An increase in exposure >1.25, was observed for products for hepatitis C (AUC 1.30; Cmax 1.26), depression (AUC 1.27), and thrombosis (Cmax 1.33). Numeric information on AUC and Cmax could not be retrieved from two dossiers, i.e. perampanel and albiglutide. In these dossiers however, the impact of sex on clearance (as PK parameter) was estimated in population PK.

**Table 2:** Women included in clinical studies evaluating pharmacokinetics (PK) in 22 Marketing Authorization Application (MAA) dossiers.

**Disease**	**MAA dossier (*N* = 22)**	**Phase I: *N* studies (women%)**	**Phase II/III: *N* studies (women%)**	**Population PK: *N* studies (women%)**
Hepatitis C	daclatasvir	25 (21)	2 (32)	2 (49)
	dasabuvir	37 (19)	0 (-)	2 (51)
	sofosbuvir	13 (29)	8 (38)	2 (37)
	simeprevir	25 (31)	7 (37)	6 (32)
	telaprevir	26 (17)	6 (40)	1 (38)
	sofosbuvir/ledipasvir	19 (31)	0 (-)	1 (39)
HIV	elvitegravir/cobicistat/emtricitabine/tenofovir alafenamide	38 (30)	2 (30)	1 (16)
	dolutegravir	27 (24)	8 (22)	1 (20)
	dolutegravir/abacavir/lamivudine	21 (51)	7 (21)	2 (16)
Depression	vortioxetine	28 (38)	0 (-)	2 (48)
Schizophrenia	loxapine	5 (47)	0 (-)	0 (-)
Epilepsy	perampanel	27 (31)	2 (43)	2 (40)
Heart failure	sacubitril/valsartan	44 (22)	9 (24)	1 (20)
Thrombosis	apixaban	26 (14)	4 (60)	1 (56)
Hypercholes-terolemia	alirocumab	9 (30)	7 (50)	1 (38)
Diabetes mellitus	albiglutide	10 (33)	9 (45)	11 (49)
	empagliflozin	29 (29)	1 (16)	1 (43)
	dulaglutide	21 (34)	7 (47)	2 (47)
	dapagliflozin	25 (17)	0 (-)	2 (51)
	canagliflozin	35 (27)	5 (55)	1 (52)
	lixisenatide	17 (35)	6 (41)	3 (49)
	alogliptin	23 (26)	4 (16)	1 (50)
**Total**		**556 (29)**	**120 (36)**	**60 (40)**

### Sex Assessment in Phase III Clinical Trials

**Figure 3:**
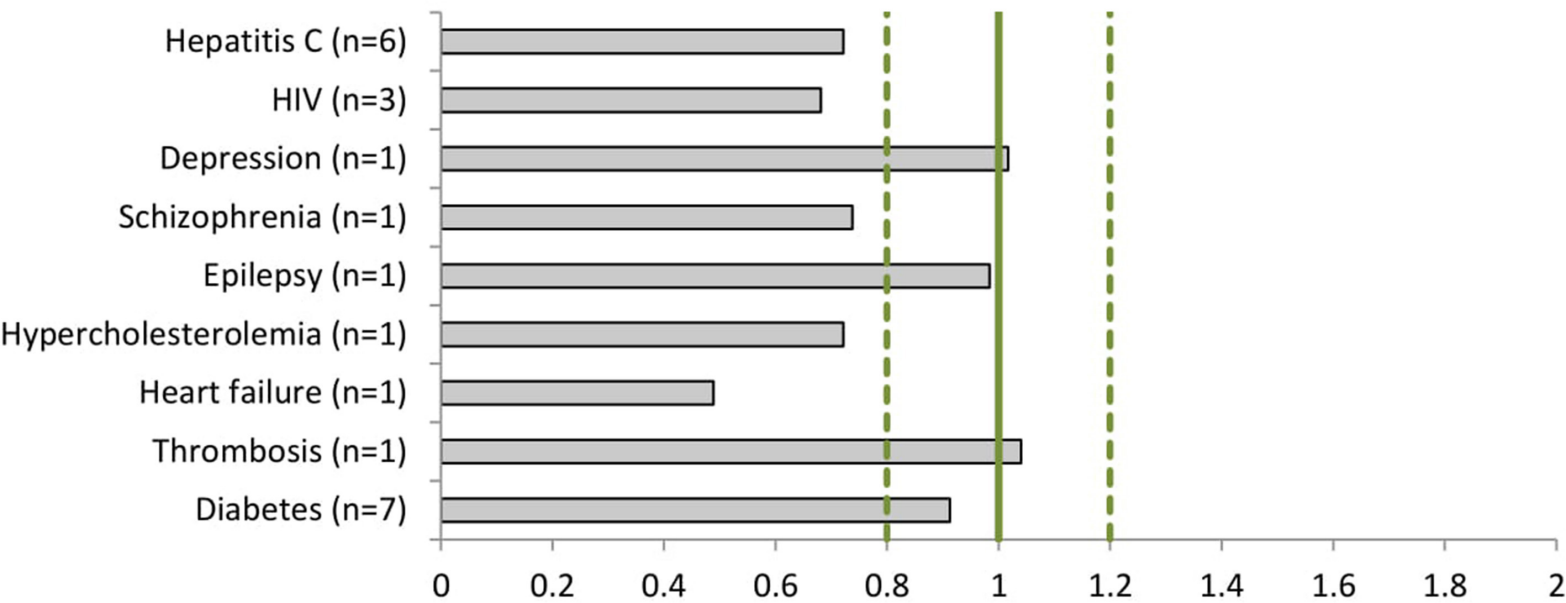
The Participation to Prevalence Ratio (PPR) of the phase III clinical studies per disease (number of dossiers included).

The dossiers contained 153 phase III clinical trials with a total of 128,507 patients, of which 52,403 (41%) were women (Table 3). All trials included both women and men. Women were represented proportionally to the disease prevalence (0.8 < PPR < 1.2) in drug dossiers in the following indications: depression (PPR: 1.02), epilepsy (PPR: 0.98), thrombosis (PPR: 1.04), and diabetes (PPR: 0.91). Women were underrepresented in the studies of hepatitis C (PPR: 0.72), HIV (PPR: 0.68), schizophrenia (PPR: 0.74), hypercholesterolemia (PPR: 0.72), and heart failure (PPR: 0.49) (Figure 3).

**Table 3:** Descriptive statistics of the inclusion of women and men in the phase III clinical trials.

**Disease**	**Drug included (*N* = 22)**	***N* Trials**	**Total population**	***N* women (%)**	***N* men (%)**
Hepatitis C	daclatasvir	1	222	145 (65)	77 (35)
	dasabuvir	6	2,315	949 (41)	1,366 (59)
	sofosbuvir	5	1,336	485 (36)	851 (64)
	simeprevir	11	2,569	854 (33)	1,715 (67)
	telaprevir	3	2,290	869 (38)	1,421 (62)
	sofosbuvir/ledipasvir	3	1,952	777 (40)	1,175 (60)
HIV	elvitegravir/cobicistat/emtricitabine/tenofovir alafenamide	5	3,465	491 (14)	2,974 (86)
	dolutegravir	4	2,667	546 (20)	2,121 (80)
	dolutegravir/abacavir/ lamivudine	12	4,299	855 (20)	3,444 (80)
Depression	vortioxetine	13	5,737	3,760 (66)	1,977 (34)
Schizophrenia	loxapine	2	658	249 (38)	409 (62)
Epilepsy	perampanel	4	2,666	1,349 (51)	1,317 (49)
Heart failure	sacubitril/valsartan	5	29,066	7,607 (26)	21,459 (74)
Thrombosis	apixaban	3	12,500	7,921 (63)	4,579 (37)
Hypercholes-terolemia	alirocumab	10	5,296	1,994 (38)	3,302 (62)
Diabetes mellitus	albiglutide	8	4,895	2,353 (48)	2,542 (52)
	empagliflozin	9	10,452	3,877 (37)	6,575 (63)
	dulaglutide	5	4,572	2,241 (49)	2,331 (51)
	dapagliflozin	12	5,662	2,729 (48)	2,933 (52)
	canagliflozin	10	7,712	3,442 (45)	4,270 (55)
	lixisenatide	8	3,507	1,874 (53)	1,633 (47)
	alogliptin	14	14,669	7,036 (48)	7,633 (52)
**Total**		**153**	**128,507**	**52,403 (41)**	**76,104 (59)**

All dossiers contained sex-specific analyses on efficacy and safety. Twenty dossiers contained numeric sex-specific information on efficacy. These data generally showed similar efficacy among women and men (Supplementary Figure 1). However, higher efficacy rates were consistently observed in women in the phase III trials of the medicinal product perampanel for the treatment of epilepsy, and there was a trend toward lower efficacy in women of the medicinal product alirocumab for the treatment of hypercholesterolemia. For some other products, an inconsistent pattern across different investigated doses or studies was shown (i.e., vortioxetine, apixaban, sofosbuvir, ledipasvir/sofosbuvir, and loxapine). The dossiers that did not contain numeric sex-specific efficacy information (i.e., dapagliflozin and telaprevir) presented other sex-specific information (i.e., *P*-value of treatment-by-sex interaction term or forest plots by sex). An additional search in the individual study reports in Module 5 showed that the sex-specific numeric information on efficacy information was presented there. For both dossiers, the efficacy was similar between women and men.

Twenty one dossiers provided numeric sex-specific information on all observed AEs. Analyses of the overall data show that a slightly higher percentage of women than men reported any AE in the treatment group (73.0 vs. 70.6%, *P* < 0.001; Table 4). Fifteen dossiers provided numeric sex-specific information about AEs in the placebo group. Again, a slightly higher percentage of women reported any AE (69.5 vs. 65.5%, *P* < 0.001; Table 4). These results were similar across the investigated medicinal products (Table 4). The dossier without numeric sex-specific information on all AEs contained numeric sex-specific information on serious AEs and AEs of special interest.

**Table 4:** Percentage of women and men experiencing any adverse drug event in **(A)** the treatment and **(B)** the placebo groups (overall and per drug).

	**Treatment**			**Placebo**		
	**Men**	**Women**	***P*-value**	**Men**	**Women**	***P*-value**
Overall	70.6	73.0	**<0.001**	65.5	69.5	**<0.001**
daclatasvir	85.0	88.0	0.256	n/a	n/a	n/a
dasabuvir	80.6	86.8	**<0.001**	72.2	82.0	0.064
sofosbuvir	87.6	94.4	**<0.001**	79.4	75.7	0.707
telaprevir	98.0	98.9	0.193	95.9	98.3	0.060
simeprevir	94.6	96.2	0.323	93.5	96.4	0.198
ledipasvir/sofosbuvir	76.8	82.8	**0.001**	n/a	n/a	n/a
elvitegravir/cobicistat/emtricitabine/ tenofovir alafenamide	84.8	82.2	0.298	n/a	n/a	n/a
dolutegravir	84.3	81.3	0.199	n/a	n/a	n/a
dolutegravir/abacavir/lamivudine	89.5	82.7	**0.032**	n/a	n/a	n/a
vortioxetine	60.2	65.5	**0.006**	54.6	61.7	**0.008**
loxapine	33.0	43.0	**0.024**	34.3	42.6	0.186
perampanel	74.9	78.8	0.136	63.6	69.4	0.202
sacubitril/Valsartan	31.0	38.3	**<0.001**	30.9	33.3	0.643
alirocumab	76.0	75.4	0.693	75.5	77.8	0.346
albiglutide	81.9	85.1	0.053	80.5	84.7	0.238
empagliflozin	66.0	73.9	**<0.001**	66.5	72.2	**<0.001**
dulaglutide	65.8	74.2	**<0.001**	63.2	70.8	0.053
dapagliflozin	59.3	64.0	**0.006**	52.4	61.6	**<0.001**
canagliflozin	56.8	62.3	**0.021**	56.0	59.0	0.443
lixisenatide	66.9	71.4	**0.026**	59.0	65.3	**0.036**
alogliptin	63.0	67.9	**0.047**	61.0	65.5	0.366

*P-values < 0.05 are considered statistically significant (bolded)*.

## Discussion

This study showed that women were included in all phases of drug development, but that their number in the phase III clinical trials is not always similar to disease prevalence rates. In the preclinical studies, female animals were included in only 9% of the PD studies, but male and female animals were included in all toxicology studies. Women were somewhat underrepresented in the PK studies (29 to 40% women) and in the phase III trials (42% women), and the representation of women in clinical studies differed across investigated diseases. All dossiers contained information on PK parameters and subgroup analyses of efficacy and safety per sex. The PK parameters generally showed a slightly higher drug exposure in women. The efficacy of the drugs was generally similar for women and men except in one dossier (perampanel) where efficacy was larger in women and one other dossier (alirocumab) where there was a trend toward lower efficacy in women. AEs were reported more frequent in women than in men in both the treatment and placebo groups.

In our study, only 9% of preclinical PD studies contained female animals. PD studies in animals are not powered to identify differences between females and males. Rather, they are intended to provide an estimate of the pharmacological dose-response effect in a disease model. There are opposing views on the importance of including (more) female animals. Some argue that inclusion of female animals is not important since the effectiveness of most drugs is similar, but others argue that both females and males should be included to understand if drug effects may be modified by potential differences in physiology and pathophysiology between both sexes (22). In line with regulatory guidelines, however, female and male animals had been included in all toxicology studies and therefore allow an adequate assessment of safety in both sexes (23). When sex differences are observed in toxicology studies, these should be justified. In general, variability/variance and differences in PK profiles are commonly underlying causes of observed sex differences in safety parameters (24). Further interrogation of potential mechanisms may be required if differences are considered clinically relevant.

A previous study using publicly available data from the FDA showed a >20% difference between the proportion of women with the disease and the proportion of women in clinical trials in 26% of the investigated drugs (10). In our study, an underrepresentation of women in phase III clinical trials was shown in five (56%) of the nine assessed diseases. Potential explanations for the difference in these proportions could be the sampled dossiers, and the use of different prevalence data.

Another study showed that the inclusion of women has improved over time, but that it is still low compared to their representativeness in the disease population (25). This is confirmed in our study investigating more recently approved drugs showing an underrepresentation of women in trials for hepatitis C, HIV, schizophrenia, hypercholesterolemia, and heart failure. Previous studies have also shown an underrepresentation of women in trials for schizophrenia (26), heart failure (19), and HIV (4). On the other hand, we found no underrepresentation for depression, epilepsy, thrombosis, and diabetes. Further studies should investigate the reasons for differences in the disproportional inclusion of women in clinical trials across diseases. A previous study conducting some exploratory analyses on the underrepresentation of women in trials of cardiovascular drugs suggested that in- and exclusion criteria might have had only a minor effect, and that the underrepresentation may have already occurred before screening (19). However, a survey study showed that women and men were to a similar extent willing to participate in clinical trials and that the few observed differences in attitudes toward trials were even more favorable among women than among men (27). This clearly demonstrates the need to further assess explanations for the disproportional inclusion of women in trials of some diseases, such as cardiovascular diseases.

Importantly, however, subgroup analyses of efficacy and safety were available per sex in all of the evaluated dossiers. It is likely that this sex-specific evaluation has improved over the years. A review of randomized controlled trials for cardiovascular disease prevention published between 1970 and 2006 showed that sex-specific analyses of the results were available in about one third of the studies (25) whereas a review of new drugs approved by the FDA between 2007 and 2009 showed that 74% of the dossiers had both efficacy and safety data presented per sex (28). The authors of that study utilized publicly available data only and may therefore have underestimated the totality of sex-specific information included in MAA dossiers.

The key question of sex differences in clinical drug trials is whether there are differences in drug response (29). Physiological differences between women and men exist, and may result in differences in the behavior of the drug in the body (30). Examples are differences in drug metabolism due to differences in body composition and concomitant use of contraceptives, resulting in different drug effects (31) or different elimination patterns as suggested with a drug like zolpidem (32). Knowledge of such sex differences is important when studying the PK of new drug molecules. Our study shows that although women are generally underrepresented in the early phase trials in which PK is evaluated, potential sex differences in critical PK parameters are well-studied. For none of the products in the three disease areas with >1.25-fold observed increases in rate and extent of exposure (Cmax, respectively, AUC), sex-specific dosing recommendations were needed. For example, the Summary of Product Characteristics (SmPC) of apixaban mentions that sex-specific analyses indicate similar drug effects (benefits and AEs) in women and men. In addition, low body weight is a criterion for lowering the dose in nonvalvular atrial fibrillation and after elective knee and hip replacement for the prevention of venous thrombotic events, and this may suffice to prevent too high exposure in—generally less heavy—women.

Detected pharmacological differences between women and men may not directly show meaningful clinical outcome differences in phase III trials, but one could also argue that they are overlooked if no sex-specific criteria are defined by regulators. Currently, standard subgroup analyses are requested by the EMA (33) but without specifying a minimal sample size of such subgroups, which is important for a reliable estimation of the variance in the population (22). In our view, MAA dossiers should thus contain phase III clinical trials with a large enough representation of women to allow identification of potential effect modification. It may not be necessary, nor realistic without inflating the trial size enormously, to power the study for efficacy in this, or for that matter in any other subgroup. It is, however, key that at the planning stage the size of these subgroups is pre-planned and reflects the population prevalence. Finally, more sex-specific information such as modification of drug effects due to hormonal status may be of relevance to premenopausal women. In an era of personalized medicine, availability of this information may guide selection and dosing of the therapy to the individual patient.

Our study showed that the efficacy of the assessed drugs was generally similar for women and men. In two dossiers, however, sex differences in efficacy were observed. At the same dosage, efficacy was higher in women for perampanel. This sex difference has been reported previously where it was suggested that it may be due to lower clearance and accompanying higher plasma concentrations in women than in men (34). For alirocumab there was a trend toward lower low density lipid cholesterol (LDL-C) reductions in women. Similar observations were made in a recent pooled analysis of 10 phase III trials (35). In both dossiers, however, when considering the totality of efficacy information available, the observed differences were not considered to change the benefit-risk balance of these products, and no differential recommendations were proposed for women vs. men in the SmPC. In the perampanel dossier the drug is titrated to therapeutic response and tolerability, and it may be that in clinical practice women receive lower maintenance doses than men.

In our study, AUC and Cmax generally were slightly higher in women than in men. This may also explain in part the observed higher number of women having AEs which is supported by post-marketing studies showing a higher number of women experiencing and/or reporting AEs (36–38). We, however, also found that AEs are more common for women in placebo groups. This suggests that there may be sex differences in nocebo effects as has been indicated previously (39).

Our data do not immediately lead to actively recommending that regulatory guidance needs to be altered with regards to inclusion of women into clinical trials in general. However, for specific diseases, more attention to including a representative sample seems desirable. For clinical practice, however, it is important that appropriate information about sex differences in efficacy and safety is made available in publicly accessible regulatory documents. Initiatives like the electronic Product Information (40) and intensified collaboration of regulators with national professional societies may facilitate translation into clinical practice and professional guidelines. Further, information on drug effects in underrepresented subgroups may be complemented by studies performed in observational data sets, i.e., real world evidence studies.

To the best of our knowledge, this is the first study that evaluated the use of female animals and participation of women across all phases of respectively, pre-clinical and clinical drug development in MAA dossiers submitted to the EMA. An important strength is that we had access to the individual study reports in the dossiers. A limitation is that a sample of dossiers across a limited number of disease areas until the year 2015 was included. The findings might not apply to other diseases, to other drugs within a therapeutic area or to more recently marketed drugs. We may have introduced selection bias, and may have overestimated the underrepresentation of women, because we selected disease areas where a number of drugs had been approved previously with a suggestion of a poorer representation of women in clinical trials (16). Also, it should be noted that we assessed the proportionality of the clinical phase III trials at a disease level. For some of the diseases we included several dossiers that generally included multiple clinical trials. This implies that for individual dossiers within a disease area representation of women could differ. Indeed, a *post-hoc* analysis shows some differences between different dossiers within the same disease area, particularly for hepatitis C (Supplementary Figure 2). This also indicates that the results of the other disease areas should be interpreted cautiously since we included only one dossier for those areas. Furthermore, for the prevalence rates we mostly used European data since we evaluated European MAA dossiers, but clinical trials are usually conducted across continents. A *post-hoc* analysis using global prevalence rates for diseases showed, however, similar results except for HIV (Supplementary Figure 3). We were not able to assess differences in recruitment of women and men across regions in the included trials. Also, the results of women to men efficacy ratios should be interpreted with caution, since these results are based on subgroup analyses and are not adjusted for possible sex differences in these subgroups, such as disease severity, comorbidity, body size, or age. This also applies to our assessment of sex differences in AEs. The analyses of sex differences in efficacy and safety were explorative. Given the large variation across the different included drugs and therapeutic areas in e.g., the studied efficacy outcomes, type of AEs, and type of analyses, future studies are required for a more detailed assessment of sex differences in the efficacy and safety of a specific drug, drug class, or therapeutic area. In these studies, the role of characteristics such as age, weight, and race should also be assessed, and there should be specific attention for differences between pre- and postmenopausal women.

## Conclusion

This study showed that women were included throughout all phases of drug development in the assessed dossiers. Although the inclusion of female animals in PD pre-clinical studies was low, female animals were included in all toxicology studies. Equally, while women were generally underrepresented in clinical studies in which PK was evaluated, all assessed dossiers contained information per sex on PK parameters. Finally, about half of the evaluated diseases did not have a proportional representation of women compared to disease prevalence rates, but a good representation was shown for some diseases, and subgroup analyses of efficacy and safety per sex were available in all evaluated dossiers. The efficacy in the assessed dossiers was generally similar for women and men, but women had slightly more often AEs both in the treatment and in the placebo groups. However, not all the information present in MAA dossiers is available for the public. Therefore, we argue that regulatory authorities should be more transparent and share these data more actively wherever possible. In certain disease areas, more attention should be paid in the planning stages of drug development to assure that a proportional group of women is included allowing a proper evaluation of potential effect modification.

## Data Availability Statement

The data were extracted from drug dossiers submitted for marketing authorization to the Dutch Medicines Evaluation Board into an Excel file. This file is stored on a secured drive of the Dutch Medicines Evaluation Board. Requests to access these datasets should be directed to Peter G. M. Mol, p.g.m.mol@umcg.nl.

## Author Contributions

All authors contributed to the development and formulation of the research question. ED-vV extracted/collected the data of the animal studies. MB extracted/collected the data of the pharmacokinetic studies. MD and ED-vV extracted/collected the data of the phase III trials. MD and SdV conducted the analyses. MD and CG-dW drafted the manuscript. SdV, CV, ED-vV, MB, PMe, IH, and PMo reviewed and edited the manuscript. All authors were involved in the analyses plan, contributed to the interpretation of the data, and have read and approved the final manuscript.

## Conflict of Interest

CG-dW is a regulatory consultant since 1-1-2019. SdV and PMo had financial support from ZonMW—The Netherlands Organization for Health Research and Development and from the European Union's Horizon 2020 research and innovation programme under the Marie Skłodowska-Curie grant agreement No 754425 for the submitted work. The remaining authors declare that the research was conducted in the absence of any commercial or financial relationships that could be construed as a potential conflict of interest.
