# Supplementary material for: Sex Proportionality in Pre-clinical and Clinical Trials: An Evaluation of 22 Marketing Authorization Application Dossiers Submitted to the European Medicines Agency
Source: Front Med (Lausanne). 2021 Mar 11;8:643028. doi: 10.3389/fmed.2021.643028 (PMC8006272; doi:10.3389/fmed.2021.643028)

Supplementary Material

Supplemental Figure 1. Women to men ratios of the efficacy of the different drugs.

1 = canagliflozin; 2 = vortioxetine; 3 = loxapine; 4 = perampanel; 5 = alirocumab; 6 = sacubitril/ valsartan; 7 = apixaban; 8 = albiglutide; 9 = empagliflozin; 10 = dulaglutide; 11 = lixisenatide; 12 = alogliptin; 13 = daclatasvir; 14 = dasabuvir; 15 = sofosbuvir; 16 = simeprevir; 17 = ledipasvir/sofosbuvir; 18 = elvitegravir/cobicistat/ emtricitabine/tenofovir alafenamide; 19 = dolutegravir; 20 = dolutegravir/abacavir/lamivudine.

= use of placebo-adjusted data. = use of active-comparator-adjusted data. =use of descriptive changes.

denotes women to men ratios in efficacy measures between 0.8 and 1.2. (It does not indicate 95% confidence intervals around the ratios as uncertainty around the efficacy parameters are not taken into account in the calculation of the women to men ratios).

Larger women to men ratios indicate larger efficacy for women except for sacubitril/valsartan (6) and apixaban (7) where larger ratios correspond with an increased risk of cardiovascular death or heart failure hospitalisation (sacubitril/valsartan) or all cause death or venous thrombotic events (apixaban) for women.

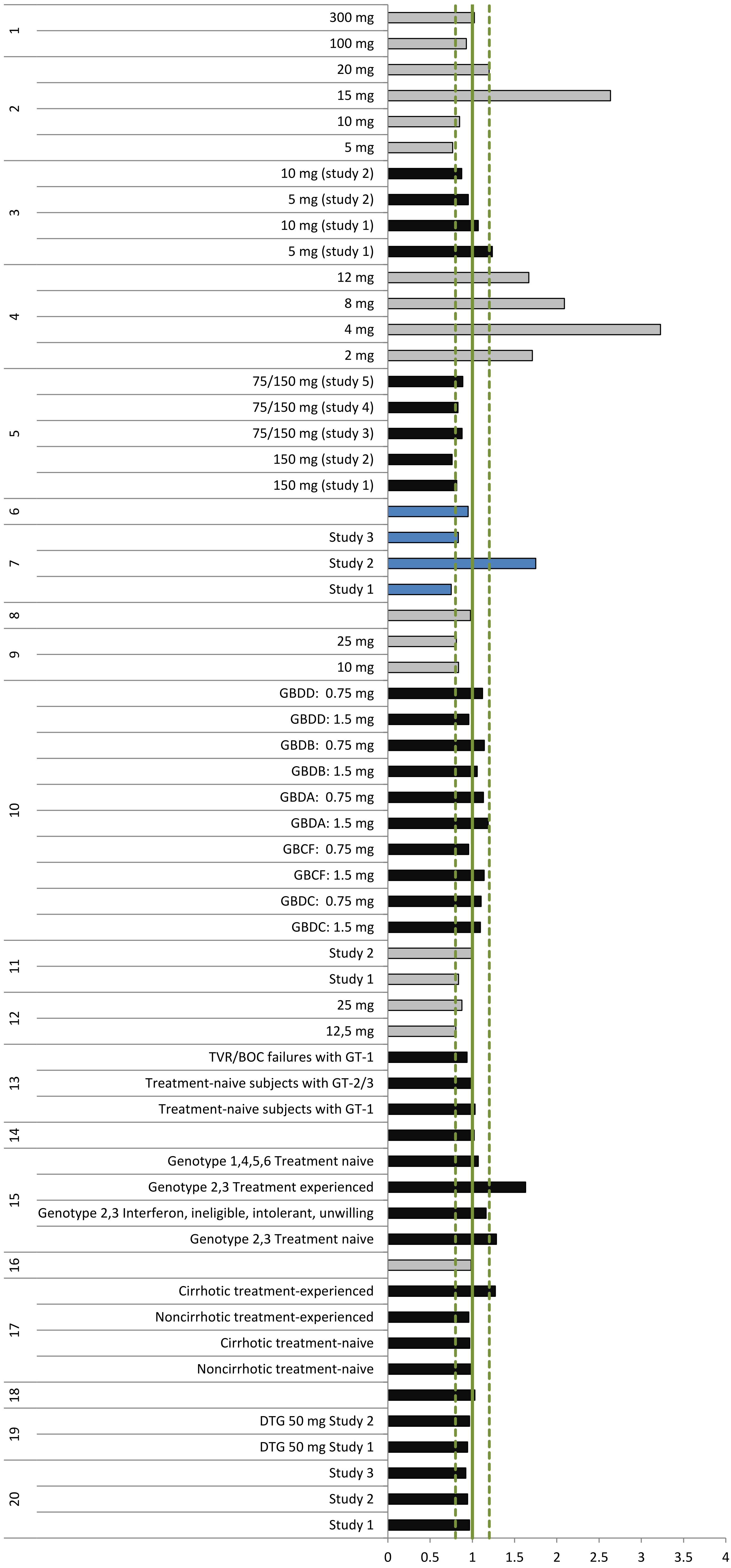

Supplemental Figure 2. The Participation to Prevalence Ratio (PPR) of the assessed drugs per disease for which more than one drug was included in the study, that is **(A)** hepatitis C, **(B)** HIV, and **(C)** diabetes. [1] ledipasvir/sofosbuvir. [2] dolutegravir/abacavir/lamivudine. [3] elvitegravir/ cobicistat/emtricitabine/tenofovir alafenamide

(A)

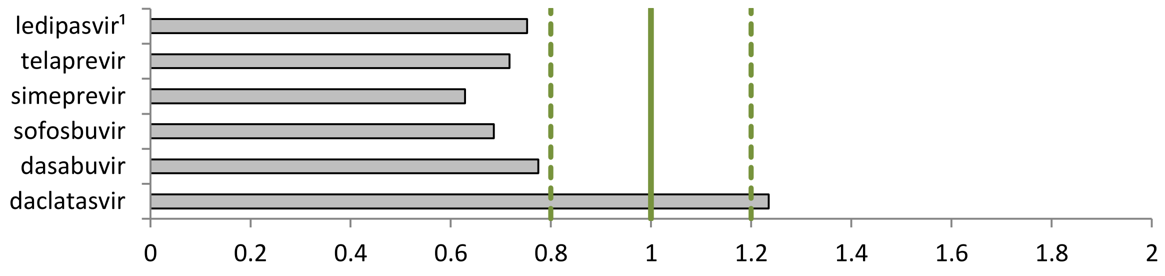

(B)

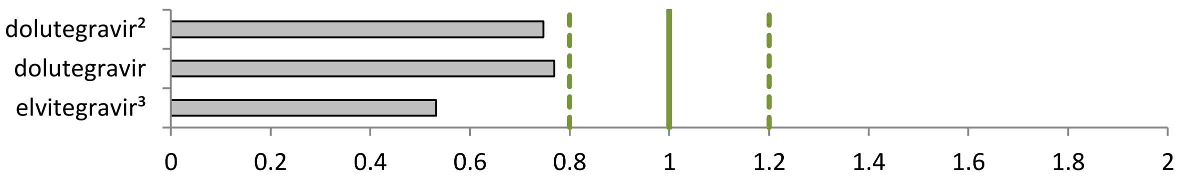

(C)

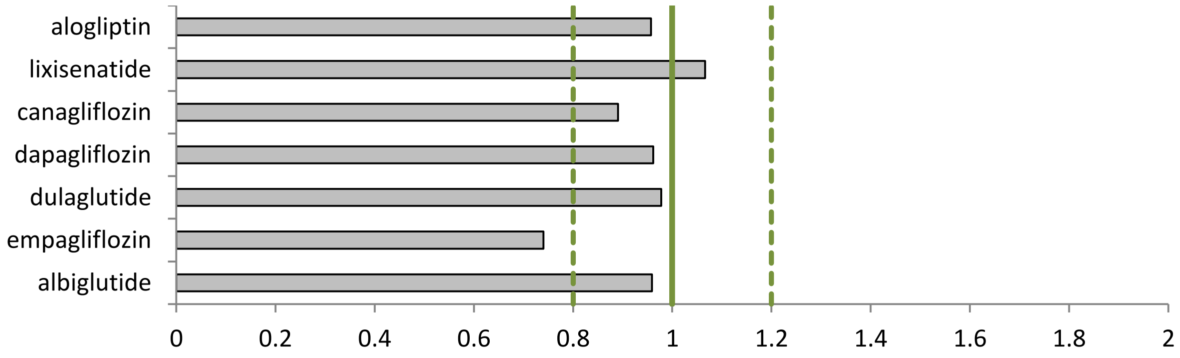

Supplemental Figure 3. The Participation to Prevalence Ratio (PPR) of the phase 3 studies per disease (number of dossiers included) using global prevalence rates based on the Global Health Data Exchange. No data are presented for thrombosis since prevalence data for this disease were obtained from scientific literature instead of from the Global Health Data Exchange.

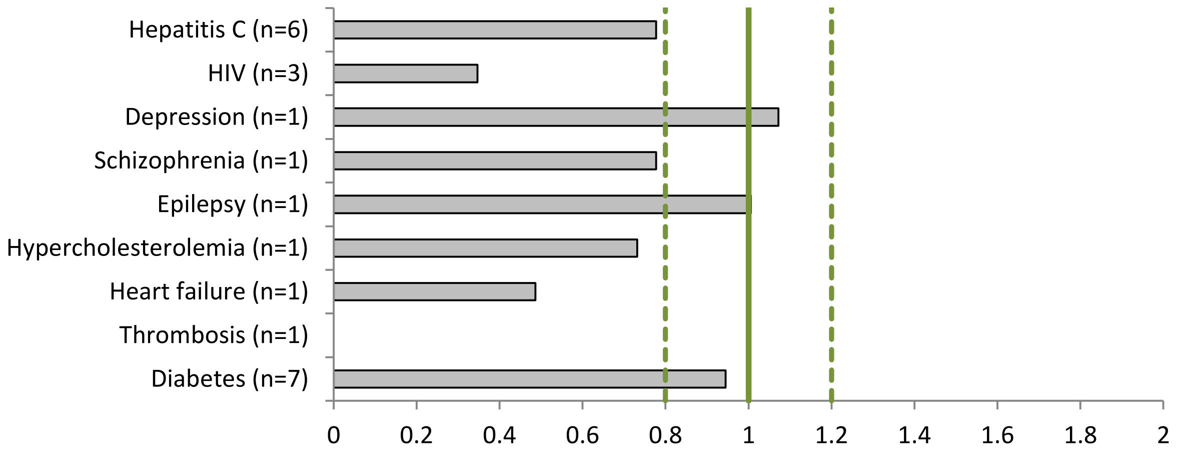

Supplement: Supplementary file 1 [file Data_Sheet_1.pdf]
